# Analytical measuring interval, linearity, and precision of serology assays for detection of SARS-CoV-2 antibodies according to CLSI guidelines

**DOI:** 10.1128/msphere.00393-24

**Published:** 2024-10-31

**Authors:** Katarzyna Haynesworth, Troy J. Kemp, Sarah A Loftus, Jordan Metz, Nicholas C. Castro, Jimmie Bullock, David Fetterer, Ligia A. Pinto

**Affiliations:** 1 Vaccine, Immunity, and Cancer Directorate, Frederick National Laboratory for Cancer Research, Leidos Biomedical Research, Inc., Frederick, Maryland, USA; Duke Human Vaccine Institute, Durham, North Carolina, USA

**Keywords:** SARS-CoV-2, Spike, Nucleocapsid, IgG, IgM, serology, enzyme-linked immunosorbent assay (ELISA), CLSI, analytical measuring interval, linearity, precision, human, serum, plasma

## Abstract

Serology testing is commonly used to evaluate the immunogenicity of COVID-19 vaccines and measure antibodies as a marker of previous infection with severe acute respiratory syndrome coronavirus 2 (SARS-CoV-2). In this study, four laboratory-developed serology enzyme-linked immunosorbent assays (SARS-CoV-2 anti-Spike and anti-Nucleocapsid immunoglobin G [IgG] and immunoglobin M [IgM]) calibrated to the WHO International Standard 20/136 were validated via analytical measuring interval (limit of blank [LOB], limit of detection [LOD], and limit of quantification [LOQ]), linearity, and precision according to the Clinical and Laboratory Standards Institute (CLSI) guidelines EP17-A2, EP06 2nd Edition, and EP05-A3. For Spike IgG, LOB was 3.0 binding antibody units per milliliter (BAU/mL), LOD was 4.1 BAU/mL, and LOQ was 27.1 BAU/mL. For Nucleocapsid IgG, LOB was 1.9 BAU/mL, LOD was 3.2 BAU/mL, and LOQ was 24.6 BAU/mL. For Spike IgM, LOB was 57.1 BAU/mL, LOD was 69.0 BAU/mL, and LOQ was 113.5 BAU/mL. For Nucleocapsid IgM, LOD was 242.2 BAU/mL, LOD was 289.9 BAU/mL, and LOQ was 572.4 BAU/mL. Each assay displayed good linearity (max % deviation from linearity (≥LOQ) = 10.7%). The result of within-run repeatability evaluation for medium positive samples was 7.7% for Spike IgG, 4.6% for Nucleocapsid IgG, 7.5% for Spike IgM, and 10.1% for Nucleocapsid IgM. The total precision, including medium positive sample variability across 20 days, three reagent kits, and two operators, was 13.5% for Spike IgG, 14.5% for Nucleocapsid IgG, 17.6% for Spike IgM, and 16.2% for Nucleocapsid IgM. The assays were successfully validated following the applicable CLSI guidelines. All assays met the ±20% deviation from linearity and the ±20% coefficient of variation specification for precision and repeatability.

**IMPORTANCE:**

Reliable and validated serology assays are of increasing importance as the severe acute respiratory syndrome coronavirus 2 (SARS-CoV-2) virus continues to evolve and cause outbreaks. Validation of serology assays along with calibration to the International and National Standards (such as anti-SARS-CoV-2 Immunoglobulin WHO International Standard 20/136 or Frederick National Laboratory for Cancer Research’s National Serology Standard COVID-NS01097) is critical to ensuring that results from clinical studies are reliable and comparable among various assays and laboratories. We describe the design and execution of a comprehensive study that established the analytical measuring intervals, linearity, precision, and repeatability of four in-house developed serology enzyme-linked immunosorbent assays (SARS-CoV-2 anti-Spike immunoglobin G [IgG] and immunoglobin M [IgM] and anti-Nucleocapsid IgG and IgM) following applicable Clinical and Laboratory Standards Institute (CLSI) guidelines. Overall, this study provides practical guidance on experimental design strategies and data analysis techniques, pertaining to the validation of COVID-19 serology assays according to CLSI guidelines, for use in clinical research studies.

## INTRODUCTION

The global spread of the novel severe acute respiratory syndrome coronavirus 2 (SARS-CoV-2) in 2019, which produced 775.3 million confirmed infections and 7.045 million deaths worldwide by 14 April 2024, resulted in a great need to determine the prevalence of infection as well as levels of immune response to the virus (1). Anti-SARS-CoV-2 antibodies, which are produced in response to viral infection and/or vaccination and measured via serological testing, may serve as an indirect marker of previous SARS-CoV-2 infection (2–4). Most patients diagnosed with COVID-19 develop detectable serum immunoglobin M (IgM) and immunoglobin G (IgG) by 4 days and peak around 7 days (IgM) and 14 days (IgG) post-symptom onset (5–11). Consequently, serology testing can be conducted post-infection to detect antibody responses against SARS-CoV-2 viral antigens such as Spike (S) protein and Nucleocapsid (N) protein (12, 13). Importantly, using these two markers, serology may help differentiate between vaccinated and infected individuals, as currently authorized COVID-19 vaccines widely used in the United States contain the Spike protein as an immunogen (i.e., a vaccinated individual will test positive for Spike antibodies and negative for Nucleocapsid antibodies) (14, 15). Meanwhile, infection with the virus, which expresses both Spike and Nucleocapsid, will induce the production of antibodies against both proteins (keeping in mind that detection and levels will vary depending on the time since infection/vaccination) (15). Thus, serology assays, such as enzyme-linked immunosorbent assay (ELISA), are valuable tools to evaluate immune responses to vaccines or infection and define longevity of antibody responses (5).

ELISA is a plate-based assay that can be utilized to identify and quantify antibodies in human sera and plasma specimens. During the assay, anti-SARS-CoV-2-specific antibodies present in clinical specimens bind to the viral protein previously coated on the plate and then are detected by a secondary enzyme-linked antibody (5). If intended for use in the clinic, COVID-19 serology assays should be optimized to ensure the reliability and validity of results by performing assay qualification and validation, including measuring parameters such as assay precision and linearity. Moreover, sensitivity and specificity should be evaluated to demonstrate that the assay meets FDA-EUA standards (>90% sensitivity, ≥93% specificity) (16–18). Finally, assays should be calibrated to International (e.g., the first or second WHO International Standard for anti-SARS-CoV-2 immunoglobulin [20/136 and 21/340, respectively] or a secondary standard such as the U.S. Human Serology Standard for Anti-SARS-CoV-2 Antibody Detection [COVID-NS01097]) to allow data harmonization and comparability of results generated by different immunoassay platforms and laboratories (19–21). The standards and guidelines developed by the Clinical and Laboratory Standards Institute (CLSI), a not-for-profit organization that develops laboratory standards worldwide, are useful tools for ensuring that assays meet all these clinical assay requirements (22).

CLSI guidelines are developed through a process meant to establish a consensus among representatives from the government, industry, and healthcare (22). Due to CLSI’s high credibility, the FDA has recognized over 100 CLSI consensus standards and guidelines, including EP05-A3 *Evaluation of Precision of Quantitative Measurement Procedures, Third Edition*, EP06 2nd Edition, *Evaluation of the Linearity of Quantitative Measurement Procedures*, and EP17-A2 *Evaluation of Detection Capability for Clinical Laboratory Measurement Procedures; Approved Guideline, Second Edition* (23–27). CLSI’s commitment to achieving global harmonization is expressed in its efforts to define common terminology to foster better communication and understanding among government, industry, and healthcare partners globally (25, 26). In this article, we describe the design and execution of a comprehensive study that established the analytical measuring intervals (AMI), linearity, precision, and repeatability of four in-house developed serology ELISA assays (SARS-CoV-2 anti-Spike IgG and IgM and anti-Nucleocapsid IgG and IgM) following applicable CLSI guidelines.

## MATERIALS AND METHODS

AMI is the range of quantities that can be measured with a specified instrumental measurement of uncertainty under defined conditions and includes the limit of blank (LOB), limit of detection (LOD), and limit of quantification (LOQ) values (27, 28). LOB is defined as the highest measurand result that is likely to be observed in a blank sample with stated probability α (25, 29). LOD is the lowest measured quantity that the test can consistently detect under routine laboratory conditions with stated probability α (25). Finally, LOQ is the lowest amount of a measurand that can be quantified with stated accuracy under the defined experimental conditions (25).

Linearity, precision, and repeatability were also evaluated for these assays. Linearity is defined as the ability to provide results that are directly proportional to the amount of measurand in the test sample, which can be verified using a linear equation (*Y* = *AX* + *B*) including a term for the *y*-intercept (27, 28). Meanwhile, the linearity interval is the range of the amount of measurand where measurement/detection adequately conforms to a fitted straight line (27). Precision refers to the closeness of agreement between measurements of quantity obtained by replicate measurements under specified conditions, usually expressed through measurements of imprecision, such as standard deviation (SD), variance, or coefficient of variation (CV) (26). Repeatability is defined as a measurement precision under specific measurement repeatability conditions, sometimes called “within-run precision” (26). Additional notes on experimental design are included in supplemental materials.

All these methods were applied to serology ligand-binding assays following guidelines outlined in CLSI EP17-A2, EP05-A3, and EP06 (25–27).

### Samples

The samples used to determine LOB were five negative plasmas collected prior to December 2019 and kindly provided by the Vitalant Research Institute. Each sample used to determine LOB was tested in duplicate over three days by one operator using two reagent lots (including two calibrator lots). The study protocol yielded 30 measurements of blank samples per reagent lot (*n* = 3 days × 5 samples × 2 replicates). The low-level samples used to test LOD were created by diluting the U.S. Human SARS-CoV-2 Serology Standard (COVID-NS01097) 90-fold (IgG assays) or 4-fold (IgM assays) in five negative plasmas. Similarly, to prepare samples used to test LOQ, COVID-NS01097 was diluted 30-fold (IgG assays) or 2-fold (IgM assays) in four negative plasmas. Table S1 contains demographic information regarding clinical samples used for linearity and precision studies. Linearity sample panels were created by serial 2-fold dilution of clinical samples in the corresponding negative matrix (sera or EDTA plasma). Precision sample panels (sera and plasma) each consisted of negative (pre-COVID), low, medium, and high antibody response samples. A low-antibody-response sample was designed to be 1.5 to 2 × LOD, the medium sample was intended to be in the middle of the AMI, and the high sample was envisioned to be close to the upper limit of the AMI. All samples were subjected to heat inactivation at 56°C for 30–60 min and then stored at −80°C until the time of testing. Our standard operating procedure, which is based on previous publications, is to heat-inactivate samples at 56°C for 30–60 min (30 min, <4-mL sample volume; 45 min, 4–10-mL sample volume; 60 min, >10-mL sample volume) (30, 31).

### Enzyme-linked immunosorbent assay

ELISA assays were conducted to measure IgG and IgM antibodies against the SARS-CoV-2 Spike [SARS-CoV-2-S(1-1208)-2P-His6] and SARS-CoV-2 Nucleocapsid (SARS-CoV-2 N [1-419], N-terminal His6 tag) proteins in human serum and plasma, using previously described protocols (32). Three lots of the Spike and Nucleocapsid proteins, obtained from the Protein Expression Laboratory at Frederick National Laboratory for Cancer Research (FNLCR), were diluted in Dulbecco’s phosphate-buffered saline (DPBS) as listed in Table S2A to D and used to coat Maxisorp 96-well plates (Thermo-Scientific Cat # 439454). The antigen coating concentrations were optimized by testing 8–17 sera from vaccinated and/or convalescent individuals along with the WHO International Standard 20/136, FNLCR’s US Serology Secondary Standard COVID-NS01097, assay standards, assay-positive controls, and assay-negative control, to ensure consistency in raw sample optical density (OD) values and binding antibody units per milliliter (BAU/mL) values.

Regarding the Spike assays (both IgG and IgM), we determined 0.15 or 0.3 µg/mL to be optimal for various Spike protein lots used in our laboratory, as was reported in our previous publications (32, 33). These antigen coating concentrations are similar to the reported optimal coating concentration (0.15 µg/mL) by Freeman et al. (34).

We evaluated Nucleocapsid protein coating concentrations within the range of 0.225–3.5 µg/mL for the IgG and IgM assays. For our IgG assay, we determined that the optimal coating concentration for all three protein lots was 0.45 µg/mL. For our IgM assay, the coating concentration showed lot-to-lot variability in the range of 0.45–3 µg/mL. These Nucleocapsid antigen coating concentrations are in a similar range to previous reports, which identified coating concentrations of 0.5 µg/mL (8, 35), 1 µg/mL (36, 37), and 2 µg/mL (38). These findings also align with Lin et al. (39), who reported that anti-IgM assays required a higher coating concentration (2 µg/mL) than anti-IgG assays (1 µg/mL) (39). The coated plates were incubated at 4°C and utilized for testing within 12–120 hours (Spike) or 32–168 hours (Nucleocapsid) post-coating. Coated antigen stability was evaluated across the minimum and maximum timeframe described for Spike (day 1 to day 5) and Nucleocapsid (day 1 to day 7) protein, which is further shown in Fig. S1. The coated Spike antigen was stable from day 1 to day 5, so this timeframe was utilized during testing. The coated Nucleocapsid antigen was stable between day 2 and day 7, so this timeframe was utilized during testing. Coated plates were washed with a PBS-Tween 20 buffer and blocked using a mixture of 4% skim milk (BD, Cat# 232100) with 0.2% Tween 20 in DPBS for 90 min. Serum-based internal standards, serum-based positive and negative controls, and clinical samples were subjected to appropriate dilution series and added to the plates, followed by incubation for 60 min (Fig. S2 shows examples of plate layouts). Next, a goat anti-human IgG enzyme horseradish peroxidase (HRP) conjugate or goat anti-human IgM HRP conjugate was added and incubated for 60 min. Three commercial lots of the IgG HRP conjugate or the IgM HRP conjugate were used, diluted in the blocking buffer as listed in Table S2A through D. After washing off the conjugate, a two-component substrate called 3,3′,5,5′-tetramethylbenzidine (Seracare, Cat# 5120-0050, three different lots) was used to initiate a color change. The plates were incubated for 25 min, and the reaction was terminated using 0.36 N sulfuric acid (J.T. Baker Cat # 4700-01, three different lots). The ELISA assays were read at 450 nanometers (nm) and 620 nm using a SpectraMax plate reader (Molecular Devices). Final background-corrected ODs were calculated as 450–620 nm. Data analysis was performed using SoftMax Pro GxP 7.0.3. The quantification of IgG and IgM was reported as binding antibody units per milliliter determined using internal standards calibrated to the anti-SARS-CoV-2 immunoglobulin WHO International Standard (20/136). Concentrations of neat internal standards are listed in Table S2A through D. A comprehensive list of reagents can be found in Table S2A through D.

### Study design and statistical analysis

The experimental study design is outlined in Table 1. Sample order randomization and statistical data analysis were conducted in SAS version 9.4 and Microsoft Excel. Graphs were generated in GraphPad Prism 10.

**TABLE 1:** Experimental design scheme^*a*^

Assay	Parameter	Measurements (*N*)	Sample set	Days of testing (*n*)	Reagent lots (*n*)	Operators (*n*)
Spike IgG	LOB	60	5 pre-COVID-19 plasmas	3	2	1
	LOD	90	5 low-level plasmas	3	2	1
	LOQ	72	4 low-level plasmas	3	2	1
	Linearity	132	1 serum and 1 plasma panels of 11 samples	1	1	1
	Precision	2,880	4 sera and 4 plasmas	20	3	2
Nucleocapsid IgG	LOB	60	5 pre-COVID-19 plasmas	3	2	1
	LOD	90	5 low-level plasmas	3	2	1
	LOQ	72	4 low-level plasmas	3	2	1
	Linearity	132	1 serum and 1 plasma panels of 11 samples	1	1	1
	Precision	2,880	4 sera and 4 plasmas	20	3	2
Spike IgM	LOB	60	5 pre-COVID-19 plasmas	3	2	1
	LOD	90	5 low-level plasmas	3	2	1
	LOQ	72	4 low-level plasmas	3	2	1
	Linearity	144	2 sera and 1 plasma panels of 8 samples	1	1	1
	Precision	2,880	4 sera and 4 plasmas	20	3	2
Nucleocapsid IgM	LOB	60	5 pre-COVID-19 plasmas	3	2	1
	LOD	90	5 low-level plasmas	3	2	1
	LOQ	72	4 low-level plasmas	3	2	1
	Linearity	144	2 sera and 1 plasma panels of 8 samples	1	1	1
	Precision	2,880	4 sera and 4 plasmas	20	3	2

^*a*^ *N* refers to the number of measurements collected for each parameter for each assay. For LOB, five plasmas collected pre-COVID were tested in duplicate over three days by one operator using two reagent lots. For LOD, five low-level plasmas were tested in triplicate over three days by one operator using two reagent lots. For LOQ, four low-level plasmas were tested in triplicate over three days by one operator using two reagent lots. For linearity, sera and EDTA plasma panels created by a serial 1:2 dilution of clinical samples in the corresponding matrix were tested by one operator using one reagent lot for each sample (tested in six replicates). For precision, four sera and four plasmas (negative, low, medium, and high analyte concentration) were tested in triplicate over 20 days by two operators using three reagent lots.

#### Limit of blank

LOB measurements were sorted from low to high (25). Pct_B_ corresponding to the desired α = 0.05 risk probability was used to calculate the rank position (25). Rank position = 0.5 + (*n* × Pct_*B*_) = 0.5 + (30 × 0.95) = 29, where *n* = 30 is the number of blank samples. The value corresponding to the 29th integral rank position was assigned as the LOB for each reagent lot (25). Since two reagent kits were used for testing, the greater of these two LOB values was assigned as the LOB for the assay. Table S3A depicts the scheme employed to determine the LOB concentrations.

#### Limit of detection

Five plasmas containing a low level of analyte were tested each in triplicate over three days by one operator using two reagent lots (including two calibrator lots). The study protocol yielded 45 measurements of low-level samples per reagent lot (*n* = 3 days × 5 samples × 3 replicates). The LOD was established statistically by means of the parametric analysis by adding the LOB to the pooled standard deviation (SD_*L*_) calculated across the low-level samples (25).


$$LOD=LOB+C_{p}SD_{L}$$

*C*_*p*_ is a multiplier to give the 95th percentile of a normal distribution (25).


$$C_{p}\;=\;\frac{1.645}{1-\frac{1}{4\left(L-J\right)}}=\frac{1.645}{1-\frac{1}{4\left(45-5\right)}}=1.655$$

*L* equals the total number of low-level sample results across all reagent lots, and *J* equals the number of unique low-level samples (25). Since two reagent lots were used for testing, the greater of these two LOD values was assigned as the LOD for the assay. An example LOD calculation for one reagent lot of Spike IgG is displayed in Table S3B.

#### Limit of quantification

Four plasma samples containing a low level of analyte were tested each in triplicate over three days by one operator using two reagent lots (including two calibrator lots). The study protocol yielded 36 measurements of low-level samples per reagent lot. The LOQ for each reagent lot was determined to be the lowest measurand concentration that can meet the accuracy specification (% bias ≤ 15%) and the precision specification (%CV ≤ 20%) (16).


$$\%\;bias=\left|\frac{reference\;-mean\;actual\;}{mean\;actual\;}\right|\;\times 100\%$$

The greater of two LOQ values were assigned as the LOQ for the assay.

#### Linearity study

Two clinical specimen matrices (serum and EDTA plasma) were used to design linearity sample panels that span the entire AMI. Due to the limited availability of clinical specimens positive for IgM, we were unable to create sample panels that reached 1,000 BAU/mL. Consequently, for the IgM assays, the internal standard serially diluted in negative serum was utilized to make the third sample panel. Each sample within the panel was tested in six replicates by one operator using one reagent lot. Six measurement replicates were plotted on the vertical (*y*) axis and the expected values on the horizontal (*x*) axis (27). The straight line reflecting the linearity of the measurement procedure was the best-fitting straight line *Y* = *AX* + *B*, where *X* is represented by the horizontal axis, *Y* is represented by the vertical axis *A*, and *B* is the *y*-intercept (27). The linear regression model analysis that corresponds to a linearity line was performed with the expected value (*E*) on the *x*-axis and measured value (mean value of six measurements) on the *y*-axis: *Y* = *A* × *E*, utilizing the MIXED Procedure statement in SAS (27). The predicted value for each sample in the sample panel was calculated using a linear regression equation (27):


predicted value=A×expected value+B,

where *B* is the *y*-intercept. Deviation from linearity for each measured value is the difference between the mean values of replicates and the best-fitted straight line (27). The allowable deviation from linearity was set at ≤20% as indicated by the FDA’s (40) 2022 Guidance for Industry (M10 Bioanalytical Method Validation and Study Sample) (40).


$$\%\;deviation=\;\left(\frac{measured-predicted}{predicted}\right)\times 100\%$$

#### Precision study

To determine the total within-laboratory precision for each assay, all covariates (between days, between lots, and between operators) were evaluated as the CV at α = 0.05. Four clinical sera samples and four clinical plasma samples were tested, each in triplicate, over 20 days by two operators using three reagent lots (including three calibrator lots). A random permutation of the sample order on plates was taken each day. The study protocol yielded 360 measurements per sample (*n* = 3 replicates/run × 20 days ×3 reagent lots × 2 operators). Our specification for % CV was not to exceed 20%, following recommendations outlined in the 2018 Bioanalytical Method Validation Guidance for Industry (41, 42).

## RESULTS

### Analytical measuring interval studies

#### Limit of blank

**Fig 1:**
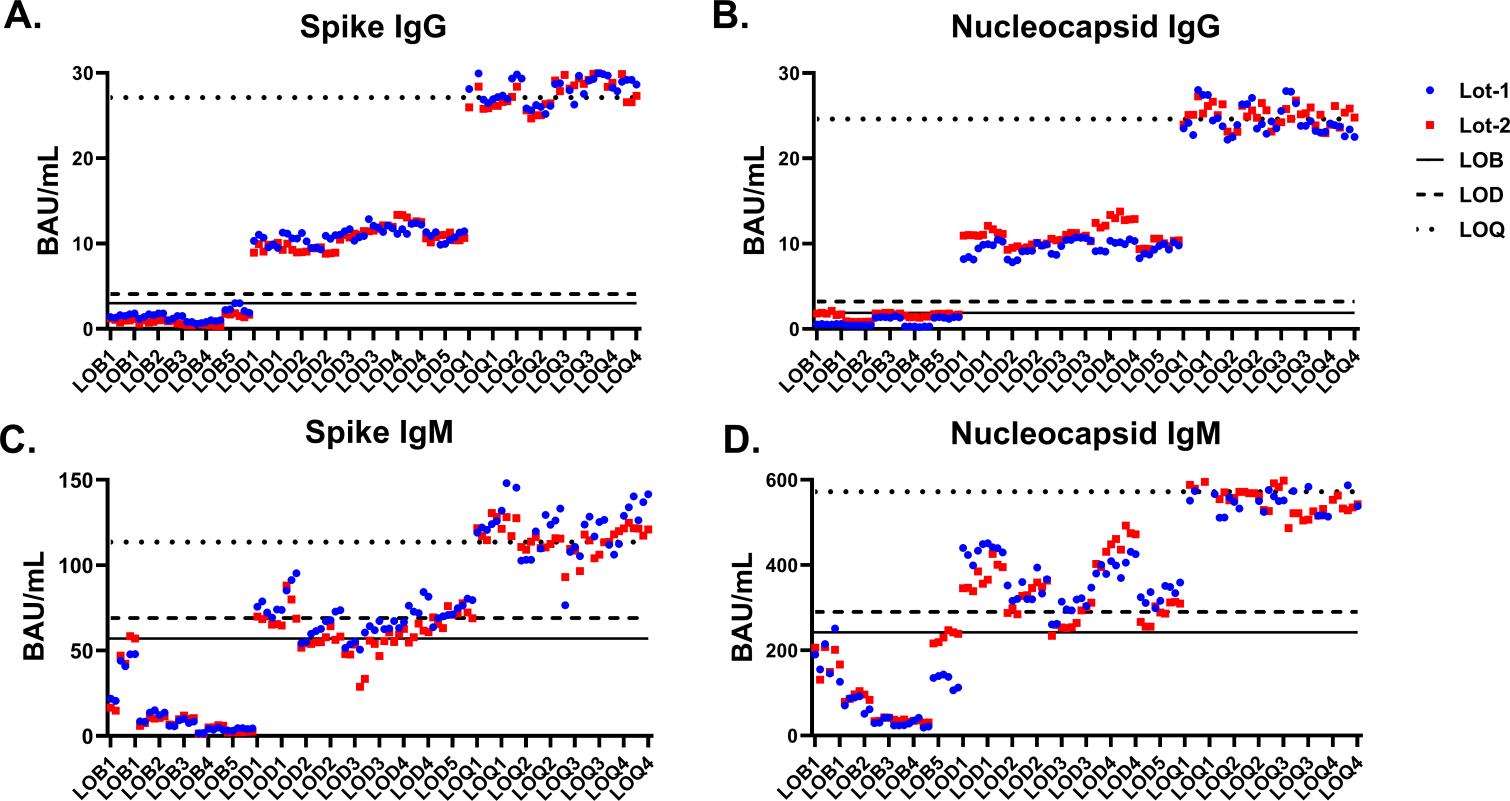
Analytical measuring intervals were determined for the four in-house antibody-binding assays. LOB, LOD, and LOQ were calculated for the in-house Spike IgG (**A**), Nucleocapsid IgG (**B**), Spike IgM (**C**), and Nucleocapsid IgM (**D**) ELISA assays.

The LOB measurements ranged 0.5–2.4 BAU/mL for Spike IgG, 0.3–1.8 BAU/mL for Nucleocapsid IgG, 2.3–39.4 BAU/mL for Spike IgM, and 28.2–232.4 BAU/mL for Nucleocapsid IgM (Table S4A). The LOB concentrations (α = 0.05) for the two reagent lots were 3.0 and 1.8 BAU/mL for Spike IgG, 1.4 and 1.9 BAU/mL for Nucleocapsid IgG, 47.8 and 57.1 BAU/mL for Spike IgM, and 214.5 and 242.2 BAU/mL for Nucleocapsid IgM, respectively. The greatest of these two values were assigned as the LOB for the assay (Fig. 1; Table 2).

**TABLE 2:** Summary of analytical measuring intervals (α = 0.05)^*a*^

AMI	Concentration (BAU/mL)			
	Spike IgG	Nucleocapsid IgG	Spike IgM	Nucleocapsid IgM
LOB	3.0	1.9	57.1	242.2
LOD	4.1	3.2	69.0	289.9
LOQ	27.1	24.6	113.5	572.4

^*a*^ AMI specifying LOB, LOD, and LOQ. Concentration expressed as BAU/mL.

#### Limit of detection

The mean concentrations of the low-level samples ranged between 9.1 and 12.6 BAU/mL for Spike IgG, 9.0 and 12.8 BAU/mL for Nucleocapsid IgG, 47.1 and 79.5 BAU/mL for Spike IgM, and 269.7 and 446.1 BAU/mL for Nucleocapsid IgM (Table S4B). The LOD concentrations (α = 0.05) for the two reagent lots were 4.1 and 3.6 BAU/mL for Spike IgG, 3.2 and 2.6 BAU/mL for Nucleocapsid IgG, 69.0 and 67.7 BAU/mL for Spike IgM, and 280.0 and 289.9 BAU/mL for Nucleocapsid IgM, respectively. The greatest of these two values were assigned as the LOD for the assay (Fig. 1; Table 2).

#### Limit of quantification

The mean LOQ concentrations ranged between 26.6 and 29.1 BAU/mL for Spike IgG, 23.3 and 25.7 BAU/mL for Nucleocapsid IgG, 107.1 and 132.0 BAU/mL for Spike IgM, and 537.9 and 599.3 BAU/mL for Nucleocapsid IgM (Table S4C). For each reagent lot, the lowest concentration that met our accuracy and precision specification (% bias ≤ 15% and %CV ≤ 20%) was assigned as the LOQ for the lot. The greatest LOQ across two reagent lots was assigned as the LOQ for the assay (Fig. 1; Table 2).

All assigned LOB values were less than the LOD values, which in turn were less than LOQ values (Fig. 1), and the signal-to-background ratio for LOD was <2. The signal-to-background ratio for LOQ was ~10 for the IgG assays and ~2 for the IgM assays. For the Spike IgG assay and the Nucleocapsid IgG assay, the LOQ was ~7 times higher than the LOD and ~40 times lower than the upper limit of quantification (ULOQ), which was assigned as 1,000 BAU/mL so as not to exceed the neat concentration of the WHO International Standard for anti-SARS-CoV-2 immunoglobulin (20/136). For the Spike IgM assay, the LOQ was ~2 times higher than the LOD and ~8 times lower than the ULOQ. For the Nucleocapsid IgM assay, the LOQ was ~2 times higher than the LOD and only ~2 times lower than the ULOQ. The claimed linear range was shortest for Nucleocapsid IgM (572.4–1,000 BAU/mL) compared to Spike IgG (27.1–1,000 BAU/mL), Nucleocapsid IgG (24.6–1,000 BAU/mL), and Spike IgM (113.5–1,000 BAU/mL) (27).

### Linearity studies

**Fig 2:**
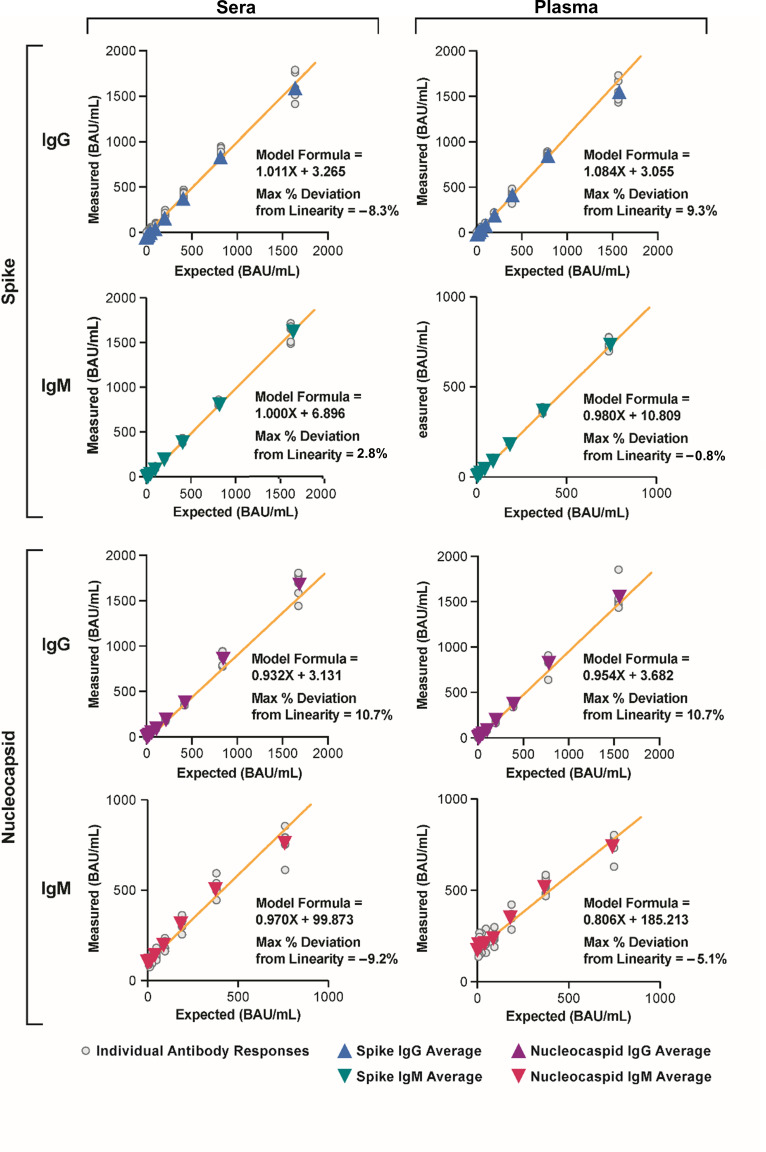
Graphs for IgG and IgM assay linearity studies. Linearity was determined for sera and plasma using the Spike IgG assay and sera and plasma using the Nucleocapsid IgG assay, and for sera, plasma, and standard using the Spike IgM and Nucleocapsid IgM assays.

For each assay, the linearity graphs are displayed in Fig. 2; Fig. S3, and deviations from linearity are recorded in Table 3; Table S5. All assays met the ±20% deviation from the linearity specification. Considering samples within the linearity interval (i.e., ≥LOQ), the absolute value maximum % deviation from linearity for Spike IgG was 8.3% (sera panel) and 9.3% (plasma EDTA panel). For Nucleocapsid IgG, the absolute value maximum % deviation from linearity was 10.7% (sera panel) and 10.7% (plasma EDTA panel). For Spike IgM, the absolute value maximum % deviation from linearity was 2.8% (sera panel), 0.8% (plasma EDTA panel), and 5.5% (standard panel). For Nucleocapsid IgM, the absolute value maximum % deviation from linearity was 9.2% (sera panel), 5.1% (plasma EDTA panel), and 10.0% (standard panel).

**TABLE 3:** Linearity studies summary^*a*^

Assay		Expected concn (BAU/mL)	Predicted value	Mean concn (BAU/mL) *N* = 6	% deviation from linearity
Spike IgG	Sera sample panel				
	1:64	25.6	29.2	30.7	5.3
	1:32	51.2	55.0	54.6	−0.8
	1:16	102.4	106.8	97.9	−8.3
	1:8	204.9	210.3	213.9	1.7
	1:4	409.7	417.4	429.1	2.8
	1:2	819.4	831.6	883.1	6.2
	Neat	1,638.8	1,659.9	1,638.8	−1.3
Spike IgG	Plasma EDTA sample panel				
	1:64	24.4	29.6	32.3	9.3
	1:32	48.9	56.1	54.5	−2.7
	1:16	97.8	109.1	98.9	−9.3
	1:8	195.6	215.1	212.4	−1.2
	1:4	391.1	427.1	434.6	1.8
	1:2	782.2	851.1	866.8	1.9
	Neat	1,564.4	1,699.0	1,564.4	−7.9
Spike IgM	Sera sample panel				
	1:16	101.1	108.0	103.7	−3.9
	1:8	202.1	209.1	215.0	2.8
	1:4	404.2	411.3	402.4	−2.2
	1:2	808.5	815.6	815.3	0.0
	Neat	1,617.0	1,624.4	1,617.0	−0.5
Spike IgM	Plasma EDTA sample panel				
	1:16	46.0	55.9	55.5	−0.6
	1:8	92.0	100.9	99.5	−1.4
	1:4	183.9	191.0	189.6	−0.8
	1:2	367.8	371.2	373.7	0.7
	Neat	735.6	731.6	735.6	0.5
Nucleocapsid IgG	Sera sample panel				
	1:64	26.2	27.5	29.1	5.5
	1:32	52.3	51.6	52.1	0.4
	1:16	104.7	100.7	95.6	−5.1
	1:8	209.3	198.3	193.8	−2.3
	1:4	418.7	393.4	382.8	−2.7
	1:2	837.3	783.7	867.7	10.7
	Neat	1,674.6	1,564.2	1,674.6	7.1
Nucleocapsid IgG	Plasma EDTA sample panel				
	1:64	24.2	26.8	29.4	9.6
	1:32	48.5	49.9	51.1	2.3
	1:16	96.9	96.1	88.5	−7.9
	1:8	193.9	188.6	197.5	4.7
	1:4	387.7	373.5	371.5	−0.5
	1:2	775.4	743.4	822.8	10.7
	Neat	1,550.8	1,483.0	1,550.8	4.6
Nucleocapsid IgM	Sera sample panel				
	1:16	47.5	146.0	143.8	−1.5
	1:8	95.0	192.0	198.9	3.6
	1:4	190.1	284.2	318.0	11.9
	1:2	380.1	468.6	505.7	7.9
	Neat	760.3	837.3	760.3	−9.2
Nucleocapsid IgM	Plasma EDTA sample panel				
	1:16	46.7	222.9	215.6	−3.2
	1:8	93.4	260.5	243.5	−6.5
	1:4	186.9	335.8	357.5	6.5
	1:2	373.8	486.4	525.5	8.1
	Neat	747.5	787.5	747.5	−5.1

^*a*^ Concentration expressed as BAU/mL.

### Precision studies

**Fig 3:**
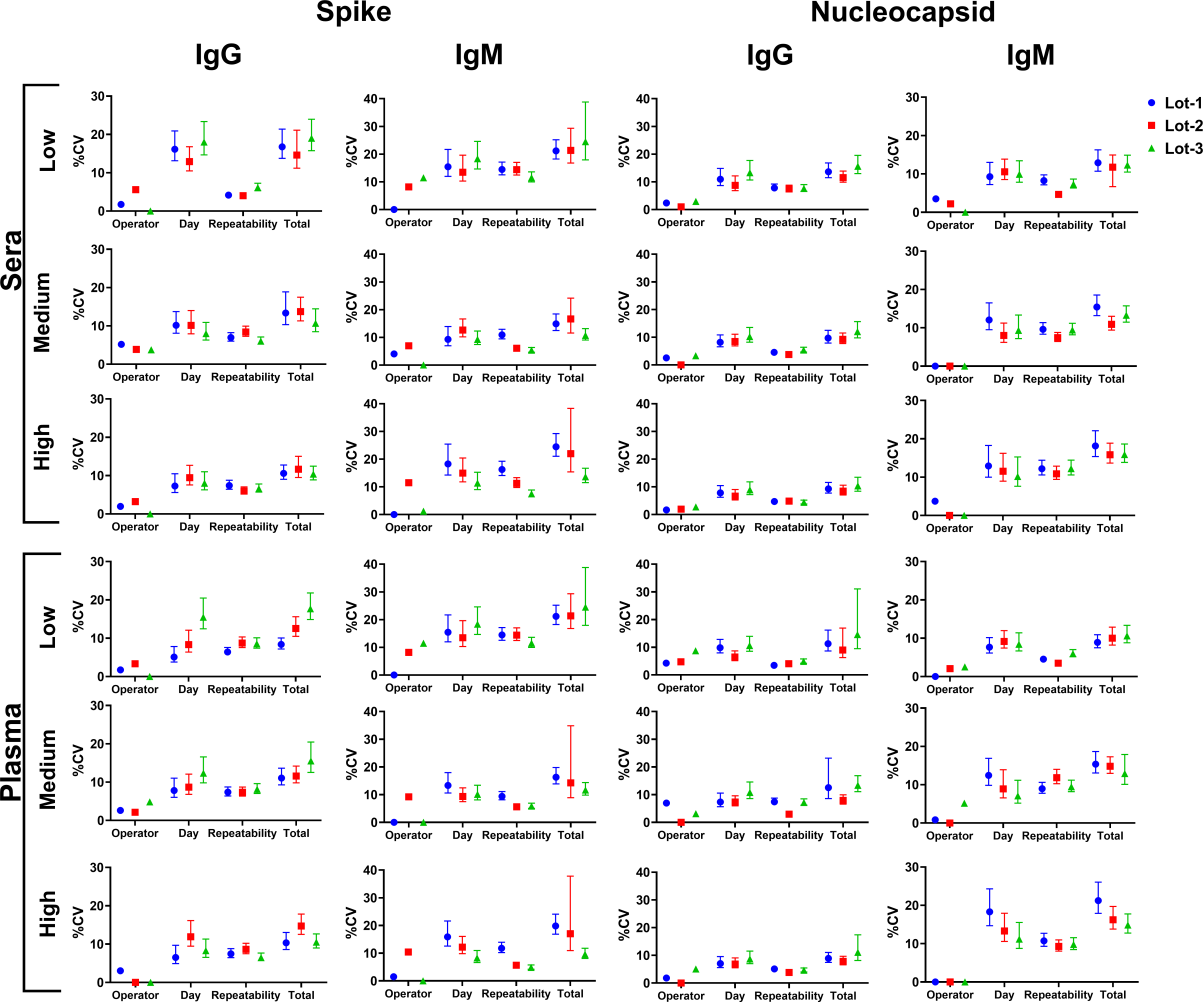
Graphical representation of the precision studies. Precision was measured using sera and plasma samples with low, medium, high antibody responses in the Spike and Nucleocapsid IgG assays and the Spike and Nucleocapsid IgM assays. Variability among two operators, across 20 days, within-run repeatability, and total precision for each reagent kit lot expressed as the %CV.

As depicted in Table 4, mean concentrations for Spike IgG low sera and plasma samples were 14.8 and 12.0 BAU/mL, medium samples were 563.3 and 656.9 BAU/mL, and high samples were 1,023.4 and 910.0 BAU/mL, respectively. Mean concentrations measured for Nucleocapsid IgG sera and plasma low samples were 5.4 and 6.2 BAU/mL, medium samples were 420.8 and 528.4 BAU/mL, and high samples were 963.2 and 890.3 BAU/mL, respectively (Table 4). Mean concentrations measured for Spike IgM sera and plasma low samples were 115.6 and 93.8 BAU/mL, medium samples were 199.9 and 273.1 BAU/mL, and high samples were 408.7 and 494.6 BAU/mL, respectively (Table 4). Mean concentrations measured for Nucleocapsid IgM sera and plasma low samples were 495.1 and 439.6 BAU/mL, medium samples were 742.3 and 679.0 BAU/mL, and high samples were 1,014.6 and 976.3 BAU/mL, respectively (Table 4). The maximum variability between the two operators was 7.9%, as observed for Spike IgM low sera sample. The variability inherent to each assay was determined based on %CV values calculated for the medium sera and plasma samples. Lot-to-lot variability did not exceed 5.4% for Spike IgG, 8.2% for Nucleocapsid IgG, 11.3% for Spike IgM, and 7.9% for Nucleocapsid IgM (Fig. 3). Within-run repeatability did not exceed 7.7% for Spike IgG, 4.6% for Nucleocapsid IgG, 7.5% for Spike IgM, and 10.1% for Nucleocapsid IgM (Table 4). Total precision that considered variability among 20 days, three reagent kits, and between two operators did not exceed 13.5% for Spike IgG, 14.5% for Nucleocapsid IgG, 17.6% for Spike IgM, and 16.2% for Nucleocapsid IgM (Table 4).

**TABLE 4:** Precision studies summary^*a*^

		Sera samples				Plasma samples			
	Assay	Negative	Low	Medium	High	Negative	Low	Medium	High
Mean concentration (BAU/mL)	Spike IgG	<4.1	14.8	563.3	1,023.4	<4.1	12.0	656.9	910.0
	Nucleocapsid IgG	<3.2	5.4	420.8	963.2	<3.2	6.2	528.4	890.3
	Spike IgM	<69.0	115.6	199.9	408.7	<69.0	93.8	273.1	494.6
	Nucleocapsid IgM	<289.9	495.1	742.3	1,014.6	<289.9	439.6	679.0	976.3
Reagent lots (%CV)	Spike IgG	NA	0.0	5.4	2.3	NA	9.8	1.6	2.4
	Nucleocapsid IgG	NA	27.6	3.8	7.6	NA	17.9	8.2	9.5
	Spike IgM	NA	5.2	11.3	16.2	NA	4.4	5.7	9.5
	Nucleocapsid IgM	NA	0.0	7.8	21.2	NA	2.0	7.9	24.3
Repeatability (%CV)	Spike IgG	NA	4.9	7.2	6.7	NA	8.1	7.7	7.6
	Nucleocapsid IgG	NA	7.8	4.6	4.7	NA	5.7	4.2	4.5
	Spike IgM	NA	13.5	7.5	11.5	NA	10.9	7.1	7.6
	Nucleocapsid IgM	NA	6.9	9.0	11.7	NA	4.8	10.1	10.0
Precision (total %CV) day/operator/kit (360 measurements/sample)	Spike IgG	NA	16.6	13.5	11.0	NA	17.1	12.9	12.2
	Nucleocapsid IgG	NA	30.6	11.0	12.1	NA	20.9	14.5	13.1
	Spike IgM	NA	23.0	17.6	25.5	NA	16.7	14.9	18.2
	Nucleocapsid IgM	NA	12.2	15.6	27.0	NA	10.0	16.2	30.1

^*a*^ Concentration expressed as BAU/mL. Precision expressed through the %CV. NA, not applicable as negative samples are below the level of detection.

## DISCUSSION

Validated serology assays are critical to generating reliable data that can support public health decisions as SARS-CoV-2 virus continues to evolve. Assay antigen selection is also critical in understanding and evaluating the antibody response to SARS-CoV-2. Here, we utilized the full-length spike and nucleocapsid proteins to evaluate anti-SARS-CoV-2 IgG and IgM antibody responses. Though neutralizing antibodies are known to target the receptor-binding domain of the spike protein (43, 44), there are additional reports highlighting that neutralizing antibodies also target other regions of the spike protein such as the NTD region of the S1 subunit, the S2 SH region, and the S2 FPs region (43). Thus, the selection of the full-length proteins was based on capturing a more comprehensive antibody response profile.

### Analytical measuring interval studies

The analytical signals for the pre-COVID blank samples detected with the Spike IgG and Nucleocapsid IgG assays were quite uniform, while the IgM assays displayed a wide range of analytical signals for the blank samples. These findings stress the importance of evaluating a comprehensive collection of blank samples in the LOB study to ensure that the assay has been fully evaluated for analytical background noise.

Overall, the hypothetical reference concentrations of all our samples used to test LOD corresponded well to the mean measured concentrations. These results support the claim that experimentally determined LOD are reliable representations of the lowest detectable concentrations with a 95% probability of being greater than the blank values. The hypothesized reference concentrations of the samples used to test LOQ also agreed with the mean measured concentration ranges. All samples used to test LOQ, except for LOQ1 for Nucleocapsid IgM (where % bias = 15.6%), met our LOQ specifications. The borderline % bias result for LOQ1 may be due to the high analytical noise that is characteristic of the Nucleocapsid IgM assay (LOB = 242.2 BAU/mL at α = 0.05).

The AMI parameters for Spike IgG determined in this study concur with published results (45). However, achieving reproducibility of results generated by different laboratories and/or when utilizing different serology assay platforms remains a challenge. Many times, in-house research and development assay results are often reported as OD or median fluorescence intensity, but such measurements produce a challenge for the comparison of results obtained across different studies (46–54). This problem would largely be addressed through the reporting of serology assay results in BAU/mL after calibration to the WHO International Standard (20-136) or secondary standards such as the U.S. Human SARS-CoV-2 Serology Standard (COVID-NS01097). Several studies have demonstrated the utility of harmonizing results from different assays or laboratories in this way (20, 55, 56). Every serology assay platform is prone to some degree of innate variability; however, calibration to a universally recognized standard can help overcome these discrepancies.

### Linearity studies

Each assay displayed good linearity above the LOQ threshold. The Nucleocapsid IgM assay displayed a significant analytical noise (LOB = 242.2 BAU/mL); however, this did not contribute to deviation from linearity. Consequently, linear regression (with a term for the *y*-intercept) appears to be a well-suited model for the evaluation of serology assay linearity. The dilution linearity study also suggests that these assays (including Nucleocapsid IgM with its short AMI range) were not prone to a hook effect, matching the requirements by the FDA’s 2022 Guidance for Industry (M10 Bioanalytical Method Validation and Study Sample) (40).

### Precision studies

A validated assay should have the capability to detect the presence of measurand through “yes/no” detection within the LOD–LOQ range and to reliably quantify measurand above the LOQ (25). In this precision study, SARS-CoV-2 anti-Spike and anti-Nucleocapsid IgG and IgM assay-specific variability measured as %CV was established using medium concentration samples. The maximum total cumulative variability over 20 days, three reagent kits, and between two operators was ≤17.6%, as it was observed for medium sera samples tested with Spike IgM. These results were comparable to other studies (57, 58). For example, Bundschuh et al. (59) reported 7.0% and 4.0% within-run variability for the IgG and IgM assays in plasma, respectively, compared to our reported %CVs of 4.2% and 10.1% in plasma, respectively (59). In addition, Bundschuh et al. (59) reported a total precision of 9.0% and 10.0% for IgG and IgM assays, compared to 14.5% for the IgG assay and 16.2% for the IgM assay in this study. Notably, our study accounted for variability across three different lots of reagents in addition to the experimental conditions described by Bundschuh et al. (59). Overall, these variability results are comparable to published results for the detection of antibodies against SARS-CoV-2 antigens in sera and plasma using ELISA (57–59).

### Conclusion

Validation of serology assays is critical to ensuring the reliability of results from research and clinical studies as well as comparability to results obtained through the same assay platforms by other groups. The CLSI has published guidance documents (such as EP17, EP06, and EP05) to promote best practices of assay validation for use by the research and clinical community (25–27). This study was designed to demonstrate a practical application of the guidelines outlined in EP17, EP06, and EP05, specifically in the validation of four ELISA assays (SARS-CoV-2 anti-Spike and anti-Nucleocapsid IgG and IgM). In addition, these results highlight how the utilization of internal standards calibrated to other recognized standards, such as WHO International Serology Standard 20/136 or FNLCR's secondary standard, US SARS-CoV-2 serology standard (COVID-NS01097), promotes the harmonization of results across various laboratories and serology assay platforms.

## SUPPLEMENTAL MATERIAL

Supplemental Material msphere.00393-24-s0001.docx Supplemental methods, Tables S1 to S5, and Figures S1 to S3.

ASM does not own the copyrights to Supplemental Material that may be linked to, or accessed through, an article. The authors have granted ASM a non-exclusive, world-wide license to publish the Supplemental Material files. Please contact the corresponding author directly for reuse.

## References

[1] World Health Organization. 2024. WHO coronavirus (COVID-19) dashboard. Available from: https://covid19.who.int. Retrieved 20 Feb 2024.

[2] Mahajan A, Manchikanti L. 2020. Value and validity of coronavirus antibody testing. Pain Phys 4S;23:S381–S390. doi:10.36076/ppj.2020/23/S381 32942795

[3] Mahmoud SA, Ganesan S, Naik S, Bissar S, Zamel IA, Warren KN, Zaher WA, Khan G. 2021. Serological assays for assessing postvaccination SARS-CoV-2 antibody response. Microbiol Spectr 9:e0073321. doi:10.1128/Spectrum.00733-21 34585943 PMC8557923

[4] Theel ES, Slev P, Wheeler S, Couturier MR, Wong SJ, Kadkhoda K. 2020. The role of antibody testing for SARS-CoV-2: is there one? J Clin Microbiol 58:e00797-20. doi:10.1128/JCM.00797-20 32350047 PMC7383527

[5] Polatoğlu I, Oncu-Oner T, Dalman I, Ozdogan S. 2023. COVID-19 in early 2023: structure, replication mechanism, variants of SARS-CoV-2, diagnostic tests, and vaccine & drug development studies. MedComm (2020) 4:e228. doi:10.1002/mco2.228 37041762 PMC10082934

[6] Renard N, Daniel S, Cayet N, Pecquet M, Raymond F, Pons S, Lupo J, Tourneur C, Pretis C, Gerez G, Blasco P, Combe M, Canova I, Lesénéchal M, Berthier F. 2021. Performance characteristics of the vidas SARS-CoV-2 IgM and IgG serological assays. J Clin Microbiol 59:e02292-20. doi:10.1128/JCM.02292-20 33419947 PMC8092742

[7] Smithgall MC, Dowlatshahi M, Spitalnik SL, Hod EA, Rai AJ. 2020. Types of assays for SARS-CoV-2 testing: a review. Lab Med 51:e59–e65. doi:10.1093/labmed/lmaa039 32657343 PMC7454768

[8] Mantus G, Nyhoff LE, Kauffman RC, Edara VV, Lai L, Floyd K, Shi P-Y, Menachery VD, Edupuganti S, Scherer EM, Kay A, McNair N, Anderson EJ, Rouphael N, Ahmed R, Suthar MS, Wrammert J. 2021. Evaluation of cellular and serological responses to acute SARS-CoV-2 infection demonstrates the functional importance of the receptor-binding domain. J Immunol 206:2605–2613. doi:10.4049/jimmunol.2001420 33952616 PMC8482837

[9] Premkumar L, Segovia-Chumbez B, Jadi R, Martinez DR, Raut R, Markmann A, Cornaby C, Bartelt L, Weiss S, Park Y, Edwards CE, Weimer E, Scherer EM, Rouphael N, Edupuganti S, Weiskopf D, Tse LV, Hou YJ, Margolis D, Sette A, Collins MH, Schmitz J, Baric RS, de Silva AM. 2020. The receptor binding domain of the viral spike protein is an immunodominant and highly specific target of antibodies in SARS-CoV-2 patients. Sci Immunol 5:eabc8413. doi:10.1126/sciimmunol.abc8413 32527802 PMC7292505

[10] Zhou C, Bu G, Sun Y, Ren C, Qu M, Gao Y, Zhu Y, Wang L, Sun L, Liu Y. 2021. Evaluation of serum IgM and IgG antibodies in COVID-19 patients by enzyme linked immunosorbent assay. J Med Virol 93:2857–2866. doi:10.1002/jmv.26741 33331654

[11] Zhao J, Yuan Q, Wang H, Liu W, Liao X, Su Y, Wang X, Yuan J, Li T, Li J, Qian S, Hong C, Wang F, Liu Y, Wang Z, He Q, Li Z, He B, Zhang T, Fu Y, Ge S, Liu L, Zhang J, Xia N, Zhang Z. 2020. Antibody responses to SARS-CoV-2 in patients with novel coronavirus disease 2019. Clin Infect Dis 71:2027–2034. doi:10.1093/cid/ciaa344 32221519 PMC7184337

[12] Hanson KE, Caliendo AM, Arias CA, Englund JA, Hayden MK, Lee MJ, Loeb M, Patel R, Altayar O, El Alayli A, Sultan S, Falck-Ytter Y, Lavergne V, Morgan RL, Murad MH, Bhimraj A, Mustafa RA. 2020. Infectious diseases society of America guidelines on the diagnosis of COVID-19: serologic testing. Clin Infect Dis:ciaa1343. doi:10.1093/cid/ciaa1343 32918466 PMC7543294

[13] Lai CKC, Lam W. 2021. Laboratory testing for the diagnosis of COVID-19. Biochem Biophys Res Commun 538:226–230. doi:10.1016/j.bbrc.2020.10.069 33139015 PMC7598306

[14] Heinz FX, Stiasny K. 2021. Distinguishing features of current COVID-19 vaccines: knowns and unknowns of antigen presentation and modes of action. NPJ Vaccines 6:104. doi:10.1038/s41541-021-00369-6 34400651 PMC8368295

[15] Plotkin S. 2022. Serologic tests for COVID-19 infections and vaccination. Pediatr Infect Dis J 41:e304–e305. doi:10.1097/INF.0000000000003574 35703283 PMC9281429

[16] Pinto LA, Shawar RM, O’Leary B, Kemp TJ, Cherry J, Thornburg N, Miller CN, Gallagher PS, Stenzel T, Schuck B, Owen SM, Kondratovich M, Satheshkumar PS, Schuh A, Lester S, Cassetti MC, Sharpless NE, Gitterman S, Lowy DR. 2022. A trans-governmental collaboration to independently evaluate SARS-CoV-2 serology assays. Microbiol Spectr 10:e0156421. doi:10.1128/spectrum.01564-21 35019677 PMC8754108

[17] Sharma S, Shrivastava S, Kausley SB, Rai B, Pandit AB. 2023. Coronavirus: a comparative analysis of detection technologies in the wake of emerging variants. Infection 51:1–19. doi:10.1007/s15010-022-01819-6 35471631 PMC9038995

[18] Harne R, Williams B, Abdelal HFM, Baldwin SL, Coler RN. 2023. SARS-CoV-2 infection and immune responses. AIMS Microbiol 9:245–276. doi:10.3934/microbiol.2023015 37091818 PMC10113164

[19] Bentley E. 2022. Establishment of the 2nd WHO international standard for anti-SARS-CoV-2 immunoglobulin and reference panel for antibodies to SARS-CoV-2 variants of concern. World Health Organization.

[20] Kemp TJ, Hempel HA, Pan Y, Roy D, Cherry J, Lowy DR, Pinto LA. 2023. Assay harmonization study to measure immune response to SARS-CoV-2 infection and vaccines: a serology methods study. Microbiol Spectr 11. doi:10.1128/spectrum.05353-22:e0535322 PMC10269912 37191544

[21] Kristiansen PA, Page M, Bernasconi V, Mattiuzzo G, Dull P, Makar K, Plotkin S, Knezevic I. 2021. WHO international standard for anti-SARS-CoV-2 immunoglobulin. Lancet 397:1347–1348. doi:10.1016/S0140-6736(21)00527-4 33770519 PMC7987302

[22] CLSI. 2024. About CLSI. Available from: https://clsi.org/about/

[23] CLSI. 2016. FDA-recognized CLSI consensus standards. Available from: https://clsi.org/

[24] FDA. 2023. Recognized consensus standards: medical devices, on us department of health and human services. Available from: https://www.accessdata.fda.gov/scripts/cdrh/cfdocs/cfStandards/results.cfm?start_search=1&sortcolumn=&productcode=&category=InVitro&type=&title=&organization=&referencenumber=®ulationnumber=&effectivedatefrom=&effectivedateto=&pagenum=100

[25] CLSI. 2012. Evaluation of detection capability for clinical laboratory measurement procedures; approved guideline. 2nd ed. CLSI EP17-A2. Clinical and Laboratory Standards Institute.

[26] CLSI. 2014. Evaluation of precision of quantitative measurement procedures; approved guideline. 3rd ed. Vol CLSI Guideline EP05-A3. Clinical and Laboratory Standards Institute.

[27] CLSI. 2020. Evaluation of linearity of quantitive measurement procedures. 2nd ed. CLSI EP06. Clinical and Laboratory Standards Institute.

[28] Killeen AA, Long T, Souers R, Styer P, Ventura CB, Klee GG. 2014. Verifying performance characteristics of quantitative analytical systems: calibration verification, linearity, and analytical measurement range. Arch Pathol Lab Med 138:1173–1181. doi:10.5858/arpa.2013-0051-CP 25171699

[29] Armbruster DA, Pry T. 2008. Limit of blank, limit of detection and limit of quantitation. Clin Biochem Rev 29 Suppl 1:S49–52. 18852857 PMC2556583

[30] Leclercq I, Batéjat C, Burguière AM, Manuguerra JC. 2014. Heat inactivation of the Middle East respiratory syndrome coronavirus. Influenza Other Respir Viruses 8:585–586. doi:10.1111/irv.12261 25074677 PMC4181824

[31] Darnell MER, Subbarao K, Feinstone SM, Taylor DR. 2004. Inactivation of the coronavirus that induces severe acute respiratory syndrome, SARS-CoV. J Virol Methods 121:85–91. doi:10.1016/j.jviromet.2004.06.006 15350737 PMC7112912

[32] Hickey TE, Kemp TJ, Bullock J, Bouk A, Metz J, Neish A, Cherry J, Lowy DR, Pinto LA. 2023. SARS-CoV-2 IgG Spike antibody levels and avidity in natural infection or following vaccination with mRNA-1273 or BNT162b2 vaccines. Hum Vaccin Immunother 19. doi:10.1080/21645515.2023.2215677:2215677 PMC10305493 37264688

[33] Bullock JL, Hickey TE, Kemp TJ, Metz J, Loftus S, Haynesworth K, Castro N, Luke BT, Lowy DR, Pinto LA. 2024. Longitudinal assessment of BNT162b2- and mRNA-1273-induced anti-SARS-CoV-2 spike IgG levels and avidity following three doses of vaccination. Vaccines (Basel) 12:516. doi:10.3390/vaccines12050516 38793767 PMC11125776

[34] Freeman B, Lester S, Mills L, Rasheed MAU, Moye S, Abiona O, Hutchinson GB, Morales-Betoulle M, Krapinunaya I, Gibbons A, Chiang C-F, Cannon D, Klena J, Johnson JA, Owen SM, Graham BS, Corbett KS, Thornburg NJ. 2020. Validation of a SARS-CoV-2 spike protein ELISA for use in contact investigations and sero-surveillance. bioRxiv. doi:10.1101/2020.04.24.057323:2020.04.24.057323

[35] Ha B, Jadhao S, Hussaini L, Gibson T, Stephens K, Salazar L, Ciric C, Taylor M, Rouphael N, Edupuganti S, Rostad CA, Tompkins SM, Anderson EJ, Anderson LJ. 2021. Evaluation of a SARS-CoV-2 capture IgM antibody assay in convalescent sera. Microbiol Spectr 9:e0045821. doi:10.1128/Spectrum.00458-21 34494855 PMC8557898

[36] Claro F, Silva D, Rodriguez M, Rangel HR, de Waard JH. 2021. Immunoglobulin G antibody response to the Sputnik V vaccine: previous SARS-CoV-2 seropositive individuals may need just one vaccine dose. Int J Infect Dis 111:261–266. doi:10.1016/j.ijid.2021.07.070 34343704 PMC8325383

[37] Horspool AM, Kieffer T, Russ BP, DeJong MA, Wolf MA, Karakiozis JM, Hickey BJ, Fagone P, Tacker DH, Bevere JR, Martinez I, Barbier M, Perrotta PL, Damron FH. 2021. Interplay of antibody and cytokine production reveals CXCL13 as a potential novel biomarker of lethal SARS-CoV-2 infection. mSphere 6:e01324-20. doi:10.1128/mSphere.01324-20 33472985 PMC7845617

[38] Boehme KW, Kennedy JL, Snowden J, Owens SM, Kouassi M, Mann RL, Paredes A, Putt C, James L, Jin J, Du R, Kirkpatrick C, Modi Z, Caid K, Young S, Zohoori N, Kothari A, Boyanton BL, Craig Forrest J. 2022. Pediatric SARS-CoV-2 seroprevalence in arkansas over the first year of the COVID-19 pandemic. J Pediatric Infect Dis Soc 11:248–256. doi:10.1093/jpids/piac010 35294550 PMC8992271

[39] Lin C-Y, Wolf J, Brice DC, Sun Y, Locke M, Cherry S, Castellaw AH, Wehenkel M, Crawford JC, Zarnitsyna VI, et al.. 2022. Pre-existing humoral immunity to human common cold coronaviruses negatively impacts the protective SARS-CoV-2 antibody response. Cell Host & Microbe 30:83–96. doi:10.1016/j.chom.2021.12.005 34965382 PMC8648673

[40] FDA. 2022. Guidance for industry: M10 bioanalytical method validation and study sample. U.S. Department of Health and Human Services.

[41] FDA. 2018. Bioanalytical method validation guidance for industry. U.S. Department of Health and Human Services.

[42] FDA. 2019. Immunogenicity testing of therapeutic protein products —developing and validating assays for anti-drug antibody detection guidance for industry. Available from: https://www.fda.gov/media/119788/download

[43] Chen Y, Zhao X, Zhou H, Zhu H, Jiang S, Wang P. 2023. Broadly neutralizing antibodies to SARS-CoV-2 and other human coronaviruses. Nat Rev Immunol 23:189–199. doi:10.1038/s41577-022-00784-3 36168054 PMC9514166

[44] Kleanthous H, Silverman JM, Makar KW, Yoon I-K, Jackson N, Vaughn DW. 2021. Scientific rationale for developing potent RBD-based vaccines targeting COVID-19. NPJ Vaccines 6:128. doi:10.1038/s41541-021-00393-6 34711846 PMC8553742

[45] Egger AE, Irsara C, Holzer B, Winkler C, Bellmann-Weiler R, Weiss G, Hartmann B, Prokop W, Hoermann G, Griesmacher A, Anliker M. 2022. Borderline and weakly positive antibody levels against the S-protein of SARS-CoV-2 exhibit limited agreement with virus neutralization titres. J Clin Virol Plus 2:100058. doi:10.1016/j.jcvp.2021.100058 35262031 PMC8651569

[46] Byrum JR, Waltari E, Janson O, Guo S-M, Folkesson J, Chhun BB, Vinden J, Ivanov IE, Forst ML, Li H, et al.. 2023. MultiSero: an open-source multiplex-ELISA platform for measuring antibody responses to infection. Pathogens 12:671. doi:10.3390/pathogens12050671 37242341 PMC10221076

[47] Larsen SE, Berube BJ, Pecor T, Cross E, Brown BP, Williams B, Johnson E, Qu P, Carter L, Wrenn S, Kepl E, Sydeman C, King NP, Baldwin SL, Coler RN. 2021. Qualification of ELISA and neutralization methodologies to measure SARS-CoV-2 humoral immunity using human clinical samples. bioRxiv:2021.07.02.450915. doi:10.1101/2021.07.02.450915 PMC8481082 34599915

[48] Mariën J, Ceulemans A, Michiels J, Heyndrickx L, Kerkhof K, Foque N, Widdowson M-A, Mortgat L, Duysburgh E, Desombere I, Jansens H, Van Esbroeck M, Ariën KK. 2021. Evaluating SARS-CoV-2 spike and nucleocapsid proteins as targets for antibody detection in severe and mild COVID-19 cases using a Luminex bead-based assay. J Virol Methods 288:114025. doi:10.1016/j.jviromet.2020.114025 33227340 PMC7678438

[49] Matefo L, Cloete van V, Armand BP, Dominique G, Samantha P, John F, Craig T, Daniel W, Theresa L, Sunetra G, Maréza B, Danelle van J, Jane BF. 2022. Validation of laboratory developed serology assays for detection of IgG antibody to severe acute respiratory syndrome coronavirus 2 in the South African population. J Virol Methods 307:114571. doi:10.1016/j.jviromet.2022.114571 35750222 PMC9212509

[50] Moncunill G, Mayor A, Santano R, Jiménez A, Vidal M, Tortajada M, Sanz S, Méndez S, Llupià A, Aguilar R, et al.. 2021. SARS-CoV-2 seroprevalence and antibody kinetics among health care workers in a Spanish hospital after 3 months of follow-up. J Infect Dis 223:62–71. doi:10.1093/infdis/jiaa696 33175145 PMC7717341

[51] Ndiaye MDB, Rasoloharimanana LT, Razafimahatratra SL, Ratovoson R, Rasolofo V, Ranaivomanana P, Raskine L, Hoffmann J, Randremanana R, Rakotosamimanana N, Schoenhals M. 2023. Using a multiplex serological assay to estimate time since SARS-CoV-2 infection and past clinical presentation in malagasy patients. Heliyon 9:e17264. doi:10.1016/j.heliyon.2023.e17264 37332901 PMC10263216

[52] Ramírez-Reveco A, Velásquez G, Aros C, Navarrete G, Villarroel-Espíndola F, Navarrete M, Fica A, Plaza A, Castro N, Verdugo C, Acosta-Jamett G, Verdugo CC. 2023. Performance estimation of two in-house ELISA assays for COVID-19 surveillance through the combined detection of anti-SARS-CoV-2 IgA, IgM, and IgG immunoglobulin isotypes. PLoS One 18:e0270388. doi:10.1371/journal.pone.0270388 36745590 PMC9901778

[53] Santano R, Barrios D, Crispi F, Crovetto F, Vidal M, Chi J, Izquierdo L, Gratacós E, Moncunill G, Dobaño C. 2021. Agreement between commercially available ELISA and in-house Luminex SARS-CoV-2 antibody immunoassays. Sci Rep 11:18984. doi:10.1038/s41598-021-98296-y 34556736 PMC8460676

[54] Tarkowski M, de Jager W, Schiuma M, Covizzi A, Lai A, Gabrieli A, Corbellino M, Bergna A, Ventura CD, Galli M, Riva A, Antinori S. 2021. Anti-SARS-CoV-2 immunoglobulin isotypes, and neutralization activity against viral variants, according to BNT162b2-vaccination and infection history. Front Immunol 12:793191. doi:10.3389/fimmu.2021.793191 34975897 PMC8718396

[55] Zhuo R, Charlton C, Plitt S, Thompson LA, Braun S, Day J, Osiowy C, Tipples G, Kanji JN. 2022. Comparison of SARS-CoV-2 spike antibody quantitative titer reporting using the World Health Organization international standard units by four commercial assays. J Clin Virol 156:105292. doi:10.1016/j.jcv.2022.105292 36108404 PMC9444336

[56] Lukaszuk K, Kiewisz J, Rozanska K, Dabrowska M, Podolak A, Jakiel G, Woclawek-Potocka I, Lukaszuk A, Rabalski L. 2021. Usefulness of IVD kits for the assessment of SARS-CoV-2 antibodies to evaluate the humoral response to vaccination. Vaccines (Basel) 9:840. doi:10.3390/vaccines9080840 34451965 PMC8402409

[57] Tré‐Hardy M, Wilmet A, Beukinga I, Favresse J, Dogné J, Douxfils J, Blairon L. 2021. Analytical and clinical validation of an ELISA for specific SARS‐CoV‐2 IgG, IgA, and IgM antibodies. J Med Virol 93:803–811. doi:10.1002/jmv.26303 32667733 PMC7405491

[58] Wilkins D, Aksyuk AA, Ruzin A, Tuffy KM, Green T, Greway R, Fikes B, Bonhomme CJ, Esser MT, Kelly EJ. 2022. Validation and performance of a multiplex serology assay to quantify antibody responses following SARS-CoV-2 infection or vaccination. Clin Transl Immunol 11:e1385. doi:10.1002/cti2.1385 PMC9040421 35495877

[59] Bundschuh C, Egger M, Wiesinger K, Gabriel C, Clodi M, Mueller T, Dieplinger B. 2020. Evaluation of the EDI enzyme linked immunosorbent assays for the detection of SARS-CoV-2 IgM and IgG antibodies in human plasma. Clin Chim Acta 509:79–82. doi:10.1016/j.cca.2020.05.047 32526218 PMC7278646

