# Supplementary material for: Analytical measuring interval, linearity, and precision of serology assays for detection of SARS-CoV-2 antibodies according to CLSI guidelines
Source: mSphere. 2024 Oct 31;9(11):e00393-24. doi: 10.1128/msphere.00393-24 (PMC11580426; doi:10.1128/msphere.00393-24)
Supplement: Supplemental Material — Supplemental methods, Tables S1 to S5, and Figures S1 to S3. [file msphere.00393-24-s0001.docx]

Analytical Measuring Interval, Linearity, and Precision of Serology Assays for Detection of SARS-CoV-2 Antibodies According to CLSI Guidelines – Supplemental Information

# Supplemental Methods

The experimental design schemes adapted from the CLSI guidance documents (EP17, EP06, or EP05) required utilization of either two or three distinct lots of reagent kits (Supplemental Table 2 A-D)^25-27^. For each serology assay, three lots of either Spike or Nucleocapsid proteins were obtained from the Protein Expression Lab at the Frederick National Laboratory for Cancer Research (FNLCR). The 3 lots of each protein originated from the same DNA sequence but were expressed and purified as three separate production batches. Qualification testing for each protein lot was performed by the COVID Serology Laboratory at FNLCR, and the coating concentration was optimized as needed. Similarly, for each serology assay, three distinct lots of internal standards were developed by the COVID Serology Laboratory at FNLCR. The internal standards consisted of pooled human serum obtained from at least two individual donors who either had an infection with the COVID-19 virus or were vaccinated with the available anti-COVID-19 vaccines. The concentration of each internal neat standard was assigned by calibrating to the First WHO International Standard for anti-SARS-CoV-2 immunoglobulin (NIBSC code: 20/136). During calibration experiments, eight-point standard curves prepared from the new internal standard and the WHO International Standard (20/136) were tested on the same plate. Based on the dose-response ranges of the WHO International Standard (20/136), combined estimates of concentrations from multiple days of testing were made, and the geometric mean concentration was assigned with a parallel line model using EDQM CombiStats (<http://www.edqm.eu/en/combistats>). Three lots of goat anti-human IgG (and anti-human IgM) enzyme horseradish peroxidase (HRP)-conjugates were purchased. Originally, IgG assays were developed using the anti-IgG secondary antibody obtained from SeraCare (Cat# 5220-0390). The CLSI guidance document suggested utilization of multiple reagent lots; however, SeraCare only offered one lot at that time. Thus, we identified Jackson ImmunoResearch (Cat# 109-036-008) as an alternative vendor. To assure equivalence, we optimized the assay concentration for the Jackson ImmunoResearch reagent to be 0.267 µg/mL for the Spike IgG assay and 0.08 µg/mL for the Nucleocapsid IgG assay. At these concentrations and for the lots tested, the Jackson ImmunoResearch anti-IgG secondary antibody can be used interchangeably with the SeraCare anti-IgG secondary antibody. Both antibodies can be used for future testing. The three lots of anti-human IgM conjugates obtained from Jackson ImmunoResearch Laboratories, Inc. displayed comparable performance characteristics observed during qualification testing performed at the COVID Serology Laboratory FNLCR. Three different lots of the remaining kit reagents were obtained from outside vendors (Supplemental Table 2 A-D). Qualification experiments were performed for each complete set of kit reagents to verify their performance in concert and to assign a dilution factor for each internal standard. During ELISA protocol execution, eight-point standard curves prepared from each internal standard by a serial 2-fold dilution in milk blocking buffer were utilized. On each plate, the internal standard dilutions were plated in duplicate along with a negative control (pooled serum), two positive controls (pooled serum), and unknown samples all plated as 4 serial 3-fold dilutions. The OD signals for the internal standard dilutions correlated to their concentrations (BAU/mL) were used to generate a 5-Parameter Logistic model that was then used to interpolate the concentrations (BAU/mL) of unknown samples located on the same plate as the internal standard. Concentration for each dilution of unknown sample was corrected by the dilution factor. The final concentration of each sample was reported as the mean concentration of each dilution that displayed linearity. On each plate, the negative control and the positive controls were required to produce concentrations (BAU/mL) within a pre-established range for each control. If either of the controls tested outside of the approved concentration range, the data was designated as invalid, and the test run was repeated. All laboratory tests were performed by a total of 4 operators. The operators received adequate training on the assay protocol by first reviewing the applicable Standard Operating Procedures (SOPs) and then successfully demonstrating competence in assay execution on three separate days while running two, four, and six 96-well plates per day respectively. The study execution was monitored by our internal Quality Unit to assure compliance with the Good Laboratory Practices (GLP) and Good Documentation Practices (GDP) rules. Quality Specialists reviewed all Data Record Forms and other documentation generated by laboratory personnel along with the raw data output files generated by the SoftMax Pro GxP 7.0.3. software.

# Supplemental Tables

**Supplemental Table 1.** Clinical Source Samples Used to Prepare Sample Panels for Precision and Linearity

**Supplemental Table 2.** Kit Reagents

1. **Spike IgG**

1. **Nucleocapsid IgG**

1. **Spike IgM**

1. **Nucleocapsid IgM**

**Supplemental Table 3.** Analytical Measuring Intervals Calculations (α = 0.05)

1. **LOB**

1. **Example LOD Calculation for Lot-1 of Spike IgG** (α = 0.05)

**Supplemental Table 4.** Analytical Measuring Intervals Summary

1. **LOB**

1. **LOD**

The U.S. Human SARS-CoV-2 Serology Standard was used to create the LOD samples, which has assigned concentrations of 764 BAU/mL, 681 BAU/mL, 246 BAU/mL, and 1037 BAU/mL respectively for Spike IgG, Nucleocapsid IgG, Spike IgM, and Nucleocapsid IgM.

Reference Value (Spike IgG, df = 90) = 764 BAU/mL ÷ 90 = 8.5 BAU/mL

Reference Value (Nucleocapsid IgG, df = 90) = 681 BAU/mL ÷90 = 7.6 BAU/mL

Reference Value (Spike IgM, df = 4) = 246 BAU/mL ÷ 4 = 61.5 BAU/mL

Reference Value (Nucleocapsid IgM, df = 4) = 1037 BAU/mL ÷ 4 = 259.3 BAU/mL

Spike IgM LOD5 sample was created by a convalescent sample 20-fold dilution in negative sample collected before December 2019.

1. **LOQ** (α = 0.05)

Green highlight indicates the lowest concentration that met our accuracy and precision specification (%Bias ≤ 15% and %CV ≤ 20%) and was assigned as the LOQ for the lot.

The U.S. Human SARS-CoV-2 Serology Standard was used to create the LOQ samples, which has assigned concentrations of 764 BAU/mL, 681 BAU/mL, 246 BAU/mL, and 1037 BAU/mL respectively for Spike IgG, Nucleocapsid IgG, Spike IgM, and Nucleocapsid IgM.

Reference Value (Spike IgG, df = 30) = 764 BAU/mL ÷ 30 = 25.5 BAU/mL

Reference Value (Nucleocapsid IgG, df = 30) = 681 BAU/mL ÷30 = 22.7 BAU/mL

Reference Value (Spike IgM, df = 2) = 246 BAU/mL ÷ 2 = 123.0 BAU/mL

Reference Value (Nucleocapsid IgM, df = 2) = 1037 BAU/mL ÷ 2 = 518.5 BAU/mL

**Supplemental Table 5.** **Linearity Studies for Internal Standard Serum Sample Panel**

# Supplemental Figures

**Supplemental Figure 1.** Stability of Coated Plates

**A:** Stability of coated Spike plates was evaluated for two protein lots (P220426.03 and P220921.03). Concentrations expressed as BAU/mL were compared at day one (Day-1) and day five (Day-5) post coating for assay low positive control and high positive control. Day-1 through Day-5 plates are used for routine testing.

**B:** Stability of coated Spike plates was evaluated for two protein lots (P220426.03 and P220921.03). Optical density values (OD450/620 nm) were compared at day one (Day-1) and day five (Day-5) post coating for assay internal standard diluted 1800-fold (STD-2) and 28800-fold (STD-6). Day-1 through Day-5 coated plates are used for routine testing.

**C:** Stability of coated Nucleocapsid (NC) plates was evaluated for assay low positive control and high positive control. Concentrations expressed as AU/mL (Arbitrary Units /milliliter) were compared at day one (Day-1), day two (Day-2), day five (Day-5), and day seven (Day-7) post coating. Day-2 through Day-7 coated plates are used for routine testing.

**D:** Stability of coated Nucleocapsid (NC) plates was evaluated for one protein lot (P200717.11). Optical Density values (OD450/620 nm) were compared at day one (Day-1), day two (Day-2), day five (Day-5), and day seven (Day-7) post coating for assay internal standard diluted 200-fold (STD-2) and 3200-fold (STD-6). Day-2 through Day-7 coated plates are used for routine testing.

**Supplemental Figure 2.** Examples of Plate Layouts

1. **Limit of Detection for Nucleocapsid IgM**

Example plate layout showing dilutions of internal standard (STD), negative control (NC), low positive control (CPL), high positive control (CPH), and five samples used to test LOD (Limit of Detection, LOD1 to LOD5) for Nucleocapsid IgM assay.

1. **Linearity for Spike IgG**

Example plate layout showing dilutions of internal standard (STD), negative control (NC), low positive control (CPL), high positive control (CPH), negative serum, and eleven linearity samples (LIN-CS-D0 to LIN-CS-D10) for Spike IgG assay.

1. **Precision for Nucleocapsid IgG**

Example plate layout showing dilutions of internal standard (STD), negative control (NC), low positive control (CPL), high positive control (CPH), and four precision samples (Low, Medium, High, and Negative) for Nucleocapsid IgG assay.

**Supplemental Figure 3.** Linearity Studies for Internal Standard Serum Sample Panel


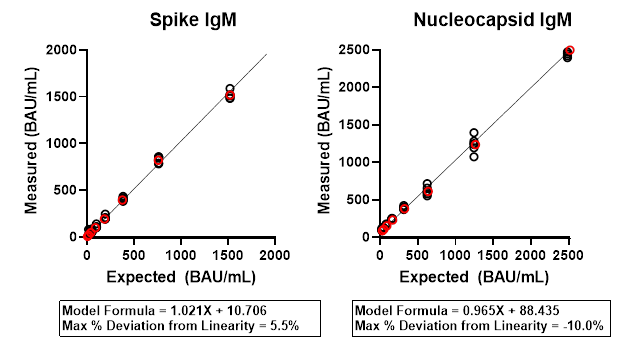


Linearity was determined for Internal Standard using the Spike IgM and Nucleocapsid IgM assays. The black circle scatter values represent individual antibody responses, red circles are IgM averages at each dilution, and the line represents the Model Formula equation.
